# An Improved Receptor-Based Pharmacophore Generation Algorithm Guided by Atomic Chemical Characteristics and Hybridization Types

**DOI:** 10.3389/fphar.2018.01463

**Published:** 2018-12-17

**Authors:** Gaoqi He, Bojie Gong, Jianqiang Li, Yiping Song, Shiliang Li, Xingjian Lu

**Affiliations:** ^1^Department of Computer Science and Engineering, East China University of Science and Technology, Shanghai, China; ^2^Shanghai Key Laboratory of New Drug Design, East China University of Science and Technology, Shanghai, China; ^3^School of Computer Science and Software Engineering, East China Normal University, Shanghai, China

**Keywords:** receptor-based pharmacophore generation, hybridization type, aromatic ring characteristic extraction, space constraint, DUD dataset testing

## Abstract

Pharmacophore-based virtual screening is an important and leading compound discovery method. However, current pharmacophore generation algorithms suffer from difficulties, such as ligand-dependent computation and massive extractive chemical features. On the basis of the features extracted by the five probes in Pocket v.3, this paper presents an improved receptor-based pharmacophore generation algorithm guided by atomic chemical characteristics and hybridization types. The algorithm works under the constraint of receptor atom hybridization types and space distance. Four chemical characteristics (H-A, H-D, and positive and negative charges) were extracted using the hybridization type of receptor atoms, and the feature point sets were merged with 3 Å space constraints. Furthermore, on the basis of the original extraction of hydrophobic characteristics, extraction of aromatic ring chemical characteristics was achieved by counting the number of aromatics, searching for residual base aromatic ring, and determining the direction of aromatic rings. Accordingly, extraction of six kinds of chemical characteristics of the pharmacophore was achieved. In view of the pharmacophore characteristics, our algorithm was compared with the existing LigandScout algorithm. The results demonstrate that the pharmacophore possessing six chemical characteristics can be characterized using our algorithm, which features fewer pharmacophore characteristics and is ligand independent. The computation of many instances from the directory of useful decoy dataset show that the active molecules and decoy molecules can be effectively differentiated through the presented method in this paper.

## Introduction

The concept of pharmacophore was firstly introduced by Ehrlich in 1909(Ehrlich, 1909). More recently, IUPAC gave an official definition as “an ensemble of steric and electronic features that is necessary to ensure the optimal supramolecular interactions with a specific biological target and to trigger (or block) its biological response”(Wermuth et al., 1998). There are two kinds of typical pharmacophore extraction methods, that is, ligand-based and structure-based (Handler and Buschmann, 2017; Machaba et al., 2017; Seidel et al., 2017). Structure-based virtual screening of pharmacophore has been developed as an important method for computer-aided drug design (Güner, 2002; Chen, 2013; Wang et al., 2017). In this case, the protein may contain ligand (holo structure) or not (apo structure). During pharmacophore extraction with holo protein structures, 4–10 different kinds of pharmacophore may be obtained due to the different strategies of conformational analysis and molecular superposition (Barnum et al., 1996). Such condition significantly reduces the efficiency of virtual screening based on pharmacophore. With the rapid progress of X-ray crystallography and nuclear magnetic resonance technologies in the determination of the protein structure of drug targets (Lam et al., 1994; Erickson et al., 2004), the pharmacophore constructed using apo protein structures becomes feasible. This method of producing a pharmacophore model directly from protein crystal structures can reveal the key elements of the receptor-ligand binding model (Böhm, 1992a).

Pocket v.2 (Chen and Lai, 2006), which was proposed by Chen and Lai at Peking University, has achieved good results by using pharmacophore extraction with apo protein structures. However, the presented pharmacophore model in Pocket v.2 only contains three kinds of chemical characteristics, which decreases the accuracy of pharmacological characteristics. In a follow-up work on Pocket v.3 (Chen et al., 2014), Chen et al. addressed its disadvantages and proposed a 5-element pharmacological characteristic set, including hydrogen-bonding acceptor (H-A), hydrogen-bond donor (H-D), positive charge (Pos), negative charge (Neg), and hydrophobic region (Hydrophobic). However, the number of chemical characteristic points extracted from Pocket v.3 is remarkably high. The points with less contribution dramatically increase the computational load of virtual screening. Thus, in this paper, we present an improved pharmacophore generation method using only the receptors based on the five kinds of probes in Pocket v.3 algorithm. Our method is guided by atomic chemical characteristics and the atomic SP hybridization (Schmitt et al., 2002; Dror et al., 2004; Shoichet et al., 2010). Atomic SP hybridization is a common way of orbital hybridization and is useful for understanding the nature of the chemical bond. This method also utilizes the constraint condition, hybrid type and space distance of receptor atoms. The major contributions of our work include the following:

1. Four kinds of chemical characteristics (H-A, H-D, Pos, and Neg) were extracted by the receptor Atomic SP hybridization, and the characteristic point set was merged under 3 Å spatial constraints.
2. On the basis of hydrophobic characteristics, the chemical characteristics of aromatic rings were extracted using the statistics number of aromatic atoms, the search for residual aromatic ring, and direction assessment of the aromatic ring axis.
3. Extraction of the pharmacophore containing six kinds of chemical characteristics was realized.

## Methods

### Flowchart of Virtual Screening of Apo Protein Structures

With the increasing number of apo protein structures, a solution must be developed for extracting pharmacophore from this type of proteins. Compared with the early work on Pocket v.2 and v.3, we propose a novel solution that aims at filtering out feature points with less pharmaceutical effect by adding aromatic features and SP hybridization form. Thus, the proposed method can reduce the quantity of pharmacophore features and improve the accuracy of virtual screening.

**Figure 1:**
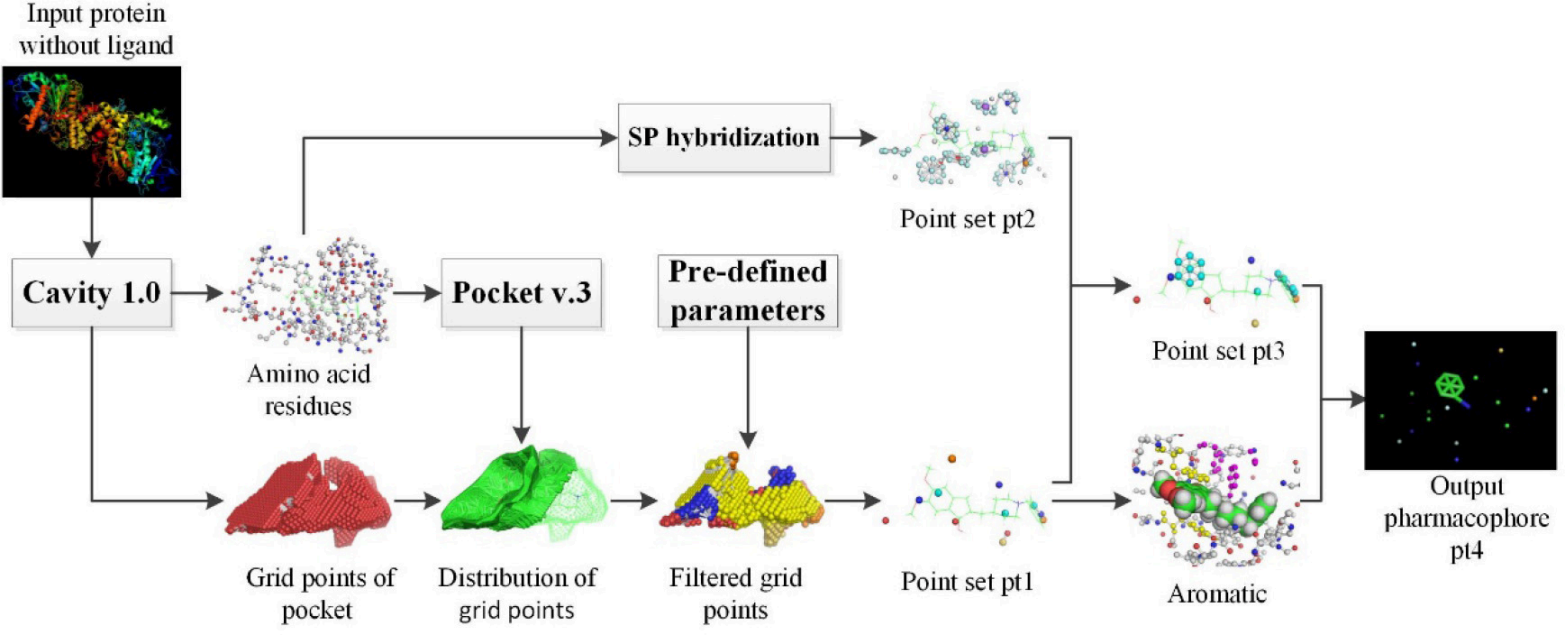
Overall flowchart of pharmacophore extraction of apo protein structures.

Figure 1 shows the overall flowchart of pharmacophore extraction with apo protein structures. Cavity 1.0(Yuan et al., 2011) was exploited to carry out the extraction of receptor pockets. Amino acid residues and grid points of pocket are the two crucial output files for the subsequent processing steps. Using Pocket v.3 software and predefined configure parameters, we get the point set pt1. On the other hand, SP hybridization technology was used to simulate the ligand characteristics of residues in the pocket to extract the pharmacophore model and we get the point set pt2. After a series of filtering operation, point set pt3 is formed. Final pharmacophore pt4 is constructed based on pt3 and extracted aromatic rings. Finally, the extracted pharmacophore models were used as input for virtual screening and tested in 28 Directory of Useful Decoy (DUD) datasets (Huang et al., 2006). The results were compared with those of LigandScout algorithm (Wolber and Langer, 2005) to verify the accuracy of the extracted pharmacological features. Default parameters were used to generate the pharmacophore model by LigandScout, which was then saved as a hypoedit file for further usage of virtual screening by the in-house PharmFit algorithm.

### Improved Pharmacophore Extraction Algorithm

#### Filtering of Pharmacophore Characteristics

Filtering of features in pharmacophore aims at removing the characteristics with less pharmaceutical effect. In our work, the energy scoring strategy used in Pocket v.3 was employed, through which the scores of the five probe atoms against the receptors were calculated accordingly. After scanning the score of each probe in each lattice point (Meng et al., 1992), we set a probe whose score is below a certain threshold to zero, because the chemical characteristics corresponding to such probe contribute less to ligand–protein binding (Carlson, 2002a,b). The threshold is set to 0.15 for HBD and HBA, 0.2 for Hydrophobic, and 0.5 for both positive and negative charges, respectively. Then, we counted the number of probes around each type of probe and determined whether it will be reserved if its statistical number is larger than a threshold value (Goodford, 1985). Here the threshold is set to 30 for HBD and HBA, 40 for Hydrophobic, and 5 for both positive and negative charges, respectively. Finally, the point with the highest score in at 2.5 Å (Chen, 2013) in a particular probe was selected as the representative chemical characteristic point of this region. All the chemical characteristic points obtained in this step were denoted as the point set pt1.

#### Extraction of Candidate Chemical Feature Point Set

**Figure 2:**
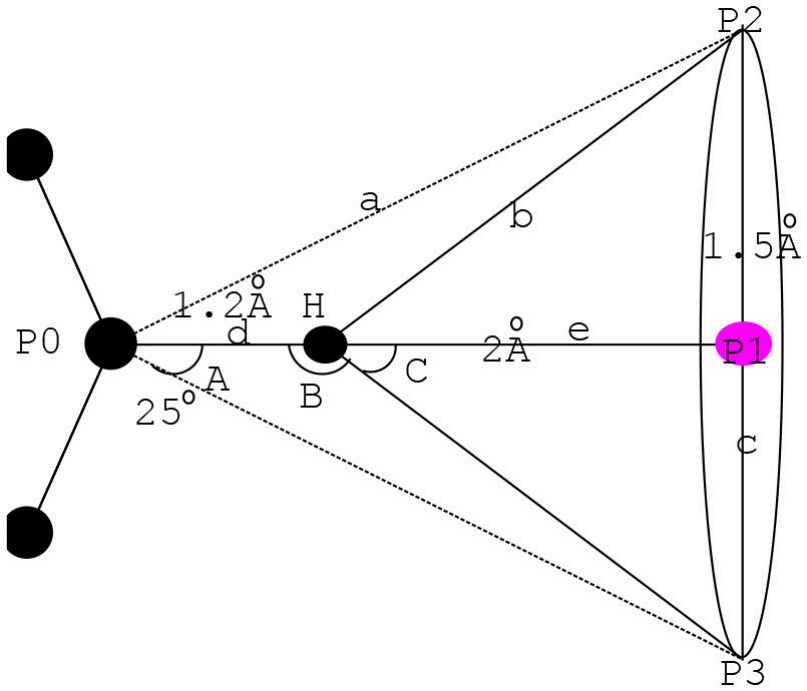
Action scope of H-A and H-D in SP2.

SP hybridization is a common way of orbital hybridization. SP hybridization is not only useful for understanding the nature of the chemical bond, but it may also be sculpted in the laboratory by means of coherent laser pulses. In this paper, the characteristics in the amino acid residue corresponding to the chemical features in the pockets were extracted by the SP hybrid model (Boobbyer et al., 1989) to form an accurate pharmacophore. Figure 2 shows the related principle with an example considering the SP2 in the SP hybrid model.

As shown in Figure 2, the center coordinates extracted by the mirror form the center point P1 at the bottom plane (Martin et al., 1993) with the radius *c*. *H* refers to the apex of the cone and serves as the action scope of H-A or H-D (Iwata and Morokuma, 1973; Taylor and Kennard, 1982).

(1) $$\begin{array}{l} {\;a}^{2}={\left(d+e\right)}^{2}+c^{2} \end{array}$$

(2) $$\begin{array}{l} \tan\left(A\right)=\frac{c}{\left(d+e\right)} \end{array}$$

(3) $$\begin{array}{l} \;e=VDW\left(H\right)+VDW\left(P1\right) \end{array}$$

In Equation 1, *d* refers to the covalent bond length of hydrogen bond. *e* is computed by Equation 3, and angle *C* is determined by the maximum feasible angle of D-H … A and A-H … D. Considering the superposition of the pharmacophore and the experience value, we obtained the maximum of c = 1.5 Å, and e = 2.0 Å. Hence, max (C) = 37° as deduced by tan (C) = *c*/*e*. Correspondingly, min (B) = 143°. The hydrogen bond will break when angle B approaches 90°. Hence, angle B must be >90°. The features in the residue corresponding to the chemical characteristics in the pockets can be extracted by the SP2 hybrid model and the distance and angle constraints. We recorded the candidate chemical feature point set obtained here as pt2.

#### Extraction of Aromatic Ring Chemical Characteristics

In our algorithm, an aromatic ring is extracted by assessing the number of aromatic atoms and the vector direction of aromatic rings. As aromatic characteristics occupy an important position in the virtual screening of pharmacophore (Rauh et al., 2004), they were added in this paper to expand the five chemical characteristics of Pocket v.3. We searched for aromatic atoms on the amino acid residue and counted the total number *n* in the 4.5 Å range of the hydrophobic chemical feature points (Böhm, 1992b; Greene et al., 1994). Then, the characteristic points with *n* ≥ 6 were kept and considered an aromatic ring. We also considered the vector direction of the aromatic ring to determine whether it can be treated as an aromatic feature. Commonly, the normal vector of aromatic rings will either be oriented (1) toward the edge or parallel to the pocket or (2) toward the inner side of the pocket. The latter exerts a more significant influence on virtual screening. Thus, aromatic rings with normal vector toward the inner side of the pocket were selected as the final aromatic features.

**Figure 3:**
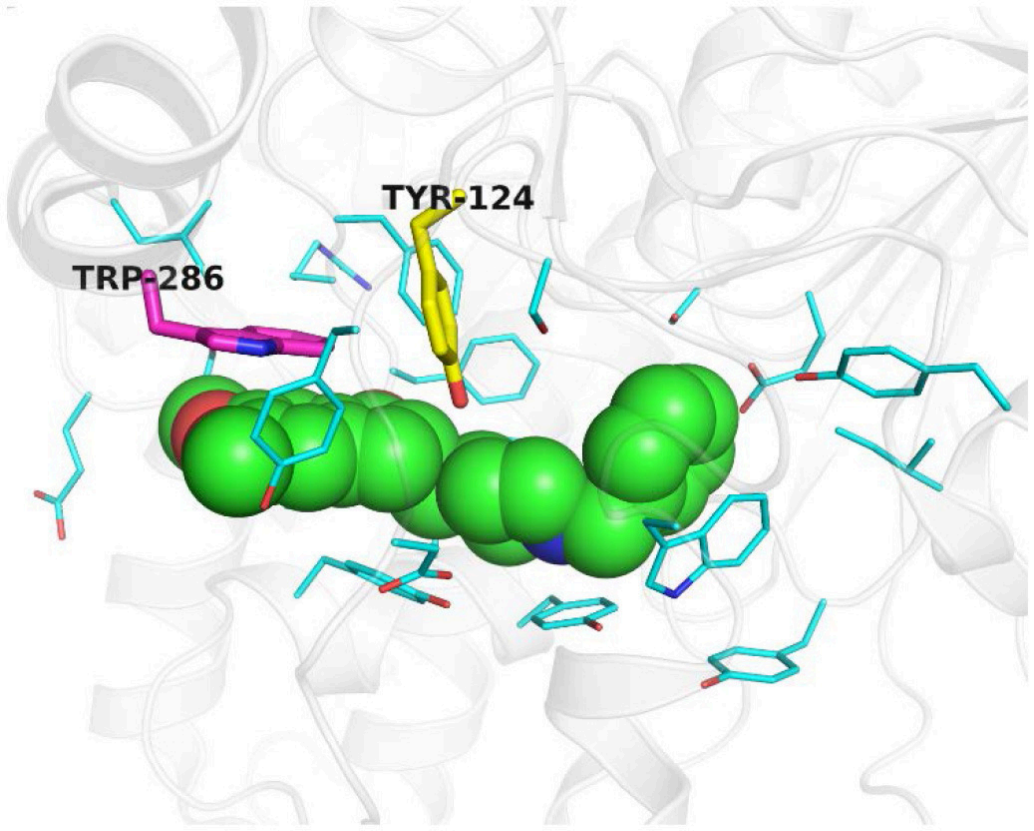
Example of aromatic rings in the binding site of ACHE (PDB ID: 4EY7). The ligand E2020 is shown as green sphere. Key residues are displayed as thin cyan sticks while key aromatic rings are highlighted as yellow and purple thick sticks. This figure is rendered by PyMol (version 1.3).

Figure 3 shows an example of aromatic rings in the binding site of ACHE (PDB ID: 4EY7). In Figure 3, the normal vector of the yellow aromatic ring is parallel to the direction of the ligand, whereas that of the purple aromatic ring heads toward the inner side of the pocket. Thus, points were extracted as an aromatic ring chemical feature when the chemical characteristic point of the hydrophobic satisfies the following: (1) the number of aromatic atoms ≥ 6; (2) the normal vector of the aromatic ring is toward the inner side of the pocket at 4.5 Å.

#### Construction of the Pharmacophore Model

**Figure 4:**
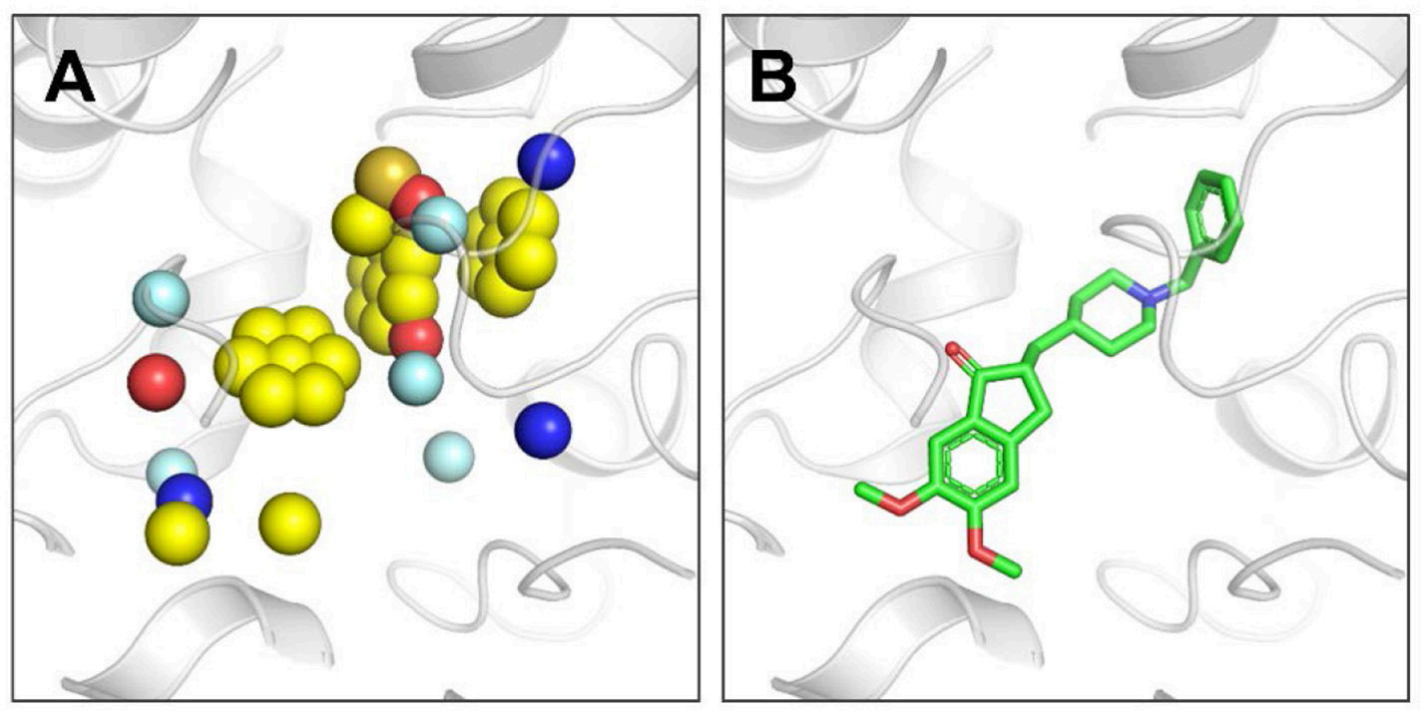
**(A)** Pharmacophores extracted by our algorithm in the ACHE binding pocket (PDB ID: 4EY7). Blue sphere represents HBD pharmacophore, red sphere represents HBA pharmacophore, single yellow sphere represents hydrophobic pharmacophore, single yellow sphere that surrounded by six planar yellow spheres represents aromatic pharmacophore, sulfur sphere represents positive, orange sphere represents negative, and palecyan sphere represents the root of h-bond. All the pharmacophore models generated by our method in this paper use this color scheme. **(B)** The bound ligand E2020 in the crystal structure of ACHE. Our algorithm generates pharmacophores independent of the bound ligand.

Through the above steps, we have obtained pt1 and pt2 by using the filtering mechanism and the SP hybrid models together with the aromatic chemical characteristics. As a result, the comprehensive pharmacophore model can be constructed. First, pt1 is handled by the SP hybrid model and then intersected with pt2 in the 3 Å range. The operation result was denoted as pt3, which contains four kinds of chemical characteristics (H-A, H-D, Pos, and Neg). Finally, hydrophobic feature in pt1, the extracted aromatic ring, and pt3 represent six important types of chemical characteristics. These characteristics make up the proposed pharmacophore model in our paper (Hoffren et al., 2001). Figure 4 displays the pharmacophores of the binding site in ACHE extracted using our algorithm independent of the bound ligand. Detailed descriptions of this pharmacophore model were in the next section.

## Results and Discussion

A series of experiments was conducted to validate the effectiveness of the presented pharmacophore extraction method.

### Comparison With Ligandscout Software

**Figure 5:**
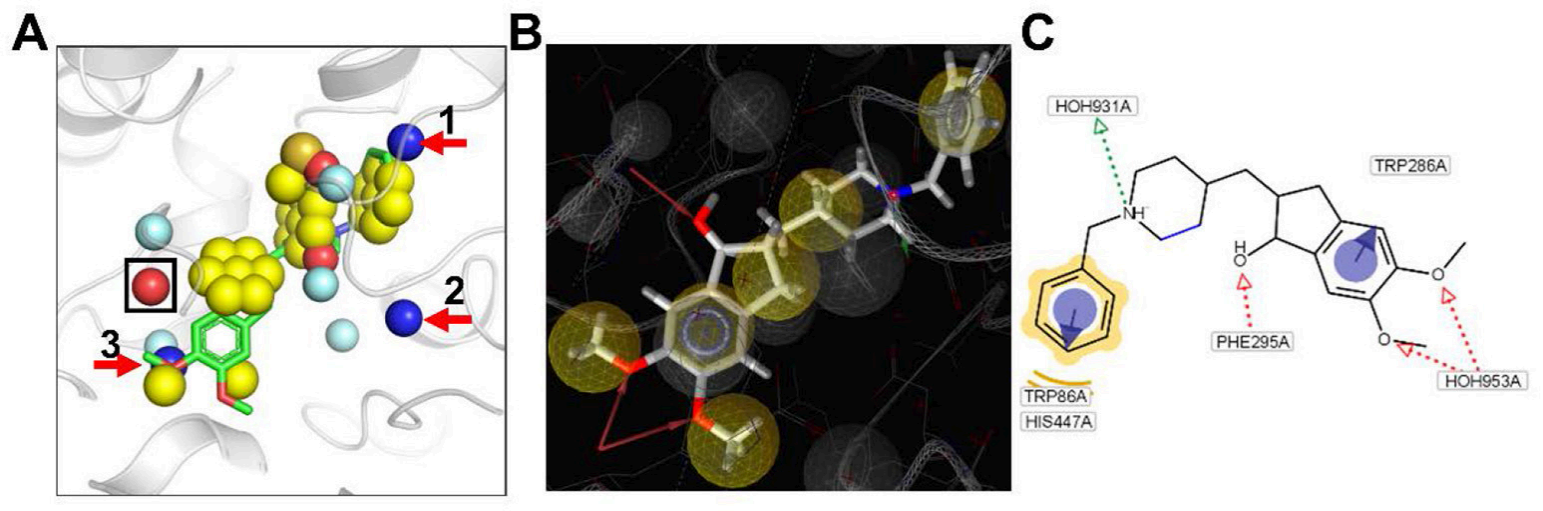
**(A)** Pharmacophores extracted by our algorithm with the bound ligand overlaid (PDB ID: 4EY7). **(B)** Pharmacophores extracted by LigandScout software. **(C)** 2D diagram for molecular interactions between E2020 and ACHE. One common HBA pharmacophore generated by both methods are highlighted by a black rectangle. Three HBD pharmacophores generated only by our method are marked by red arrows.

The pharmacophore model of ACHE was also generated through LigandScout software using the PDB code of 4EY7 (Figure 5). The comparison of the pharmacophore model generated by our method and LigandScout was illustrated in Figure 5.

#### Analysis 1: Comparison of the Distribution of Chemical Characteristic Points Corresponding to Pharmacophores

Figure 5A displayed the pharmacophore model generated by our method for ACHE with the ligand E2020 displayed only for the convenience of analysis. There were three HBA pharmacophores predicted by our method, and the one marked by black rectangle corresponds to the HBA pharmacophore generated from the hydroxyl group of E2020 by LigandScout. The proposed algorithm is independent of ligand information; such condition is necessary for the LigandScout algorithm. However, these two algorithms feature corresponding chemical characteristic of HBA, because both use the SP hybrid concept of atoms. In addition, there were five hydrophobic pharmacophores predicted by our method, and three of them are aromatic. By overlaying the bound ligand E2020 to the pharmacophore model generated by our method, it could be obviously observed that the two hydrophobic pharmacophores around the two methoxy groups and the two aromatic pharmacophores around the two benzene rings in E2020 are all accurately predicted by our method. There was another hydrophobic center including an aromatic feature around the piperidine ring predicted by our method while LigandScout predicted one hydrophobic feature at that place. Collectively, the pharmacophores predicted by our method can match most of the pharmacophores predicted by LigandScout.

#### Analysis 2: Comparison of Different Chemical Characteristic Points of Pharmacophores

Apart from the above common pharmacophores, our method predicted two more HBA, three HBD, and one Neg center compared with LigandScout. Our method failed to predict the Pos pharmacophore around the charged nitrogen atom of the piperidine ring which directly formed one hydrogen bond with HOH931 other than an amino acid residue of the protein as displayed in Figures 5B,C. The above differences between the pharmacophore models generated by our method and LigandScout mainly due to the reason that our method predicts pharmacophores using the apo protein structure and does not consider water molecules.

**Figure 6:**
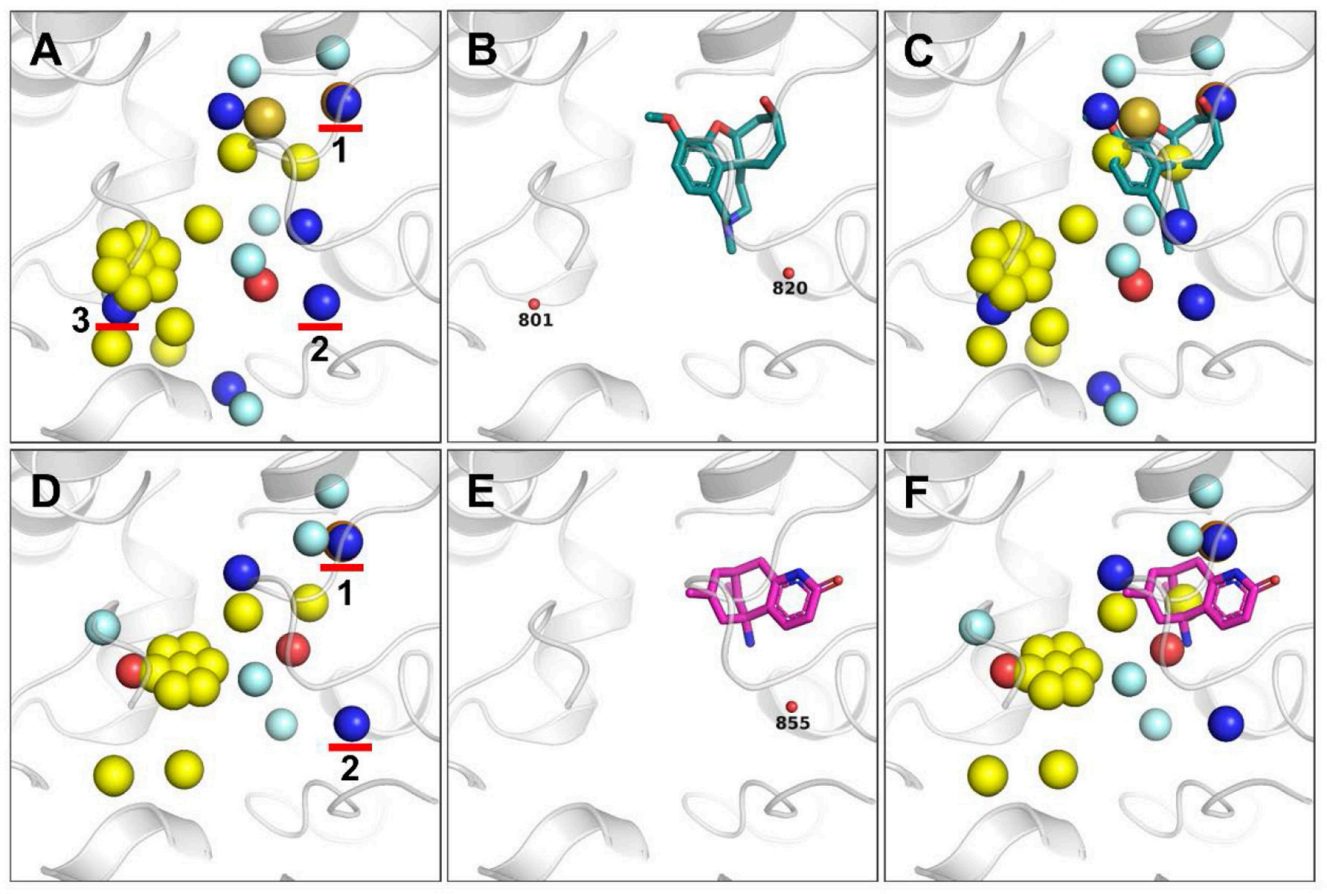
Pharmacophores generated based on the crystal structures of ACHE with PDB codes of 4EY6 **(A–C)** and 4EY5 **(D–F)**. Three HBD pharmacophores generated only by our method are marked by red arrows **(A,D)**. The ligand (-)-galanthamine bound in 4EY6 is displayed as green sticks **(B,C)**, while ligand (-)-huperzine A bound in 4EY5 is displayed as magenta sticks **(E,F)**. Water molecules are shown as small red balls **(B,E)**.

To validate whether the additional pharmacophores extracts by our method appear in other crystal structures of ACHE, we calculated pharmacophores on another two structures of ACHE with PDB codes of 4EY6 and 4EY5. For both cases of 4EY6 and 4EY5 (Figures 6A–F), the five hydrophobic pharmacophores in Figure 5A were also extracted, indicating that those pharmacophores are important for ligand binding. In addition, two of the three HBD pharmacophores in Figure 5A were also extracted simultaneously for 4EY6 and 4EY5, suggesting that HBD pharmacophore 1 and 2 highlighted in Figures 6A,C are important pharmacophores. By overlaying the bound ligand in 4EY6 and 4EY5 (Figures 6B,E) with the respective pharmacophore model (Figures 6C,F), we found that except for the well-matched hydrophobic pharmacophore, both of the ligands have a hydrogen bond donor around the HBD pharmacophore 1: (-)-galanthamine bound in 4EY6 has a hydroxy group as HBD while (-)-huperzine A bound in 4EY5 has an amine group in the amide as HBD. Although no ligand atoms bind at the places around HBD pharmacophore 2 and 3, we still found crystal water molecules as HBD. For the place around HBD pharmacophore 2, we found water molecules W820 and W855 bound at that place as HBD in 4EY6 and 4EY5, respectively (Figures 6B,E). For the place around HBD pharmacophore 3, we found one water molecule W801 bound at that place as HBD in 4EY6 (Figure 6B). The results suggested that those HBD features can be considered in the drug discovery and design pipeline against ACHE. Those results directly proved that our ligand-independent pharmacophore extraction method is reliable and superior to LigandScout in detecting pharmacophores where no ligand atoms bind. The interactions between the receptor and ligand should be evaluated when the LigandScout algorithm extracts pharmacophores. Only when interaction exists will the corresponding chemical characteristics be extracted. When a ligand is determined, only one type of interaction with the receptor exists in the static model. However, the actual interaction between the receptor and the ligand is a dynamic process. Our ligand-independent pharmacophore extraction method can adapt to the conformational changes of the receptor. Consequently, the proposed ligand-independent pharmacophore extraction method shows its advantages against LigandScout in terms of pharmacophore extraction, and can be helpful for pharmacophore-based virtual screening to find more diverse active molecules.

### Verifying the Feasibility of Using DUD Datasets

In the experiment, we executed both the proposed algorithm and LigandScout algorithm to produce the corresponding pharmacophore model by using 28 DUD datasets. These datasets include ACHE, COX2, PR, P38, PNP, Inha, EGFR, TK, FGFR1, COX1, PDE5, Rxra, GR, HSP90, AR, thrombin, CDK2, MR, ADA, FXA, PPARG, GPB, SRC, DHFR, ACE, VEGFR2, ER-Agonist, and HIVPR.

The feasibility of the pharmacophore model extracted by the proposed algorithm was verified by the in-house PharmFit algorithm. PharmFit uses a triangular hashing algorithm to realize the pharmacophore superimposition with high efficiency and adopts the default parameter setting. Xu et al. used PharmFit for virtual screening in their work of CavityPlus (Xu et al., 2018). Hence, we scored the activity and the decoy molecule of each dataset by using PharmFit. Then, the area under the receiver operating characteristic curve (AUC) and the enrichment factors (EF)(Machaba et al., 2017) were calculated. EFs are used in virtual screening to distinguish the capability between active and inactive molecules and is defined by Equation 4 (Chen, 2013). Here, the EF values were computed under 1, 2, and 5% filter libraries.

(4) $$\begin{array}{l} EF=\frac{Hits_{sampled}}{N_{sampled}}\times \frac{N_{total}}{Hits_{total}} \end{array}$$

where *Hits*_*sampled*_ represents the number of hit active molecules in the corresponding *x*% filter Library, *N*_*sampled*_ corresponds to the total number of molecules, *N*_*total*_ denotes the total number of molecules, and *Hits*_*total*_ stands for the total number of active molecules in the filter library.

#### Comparison of AUC Using 28 Datasets

**Figure 7:**
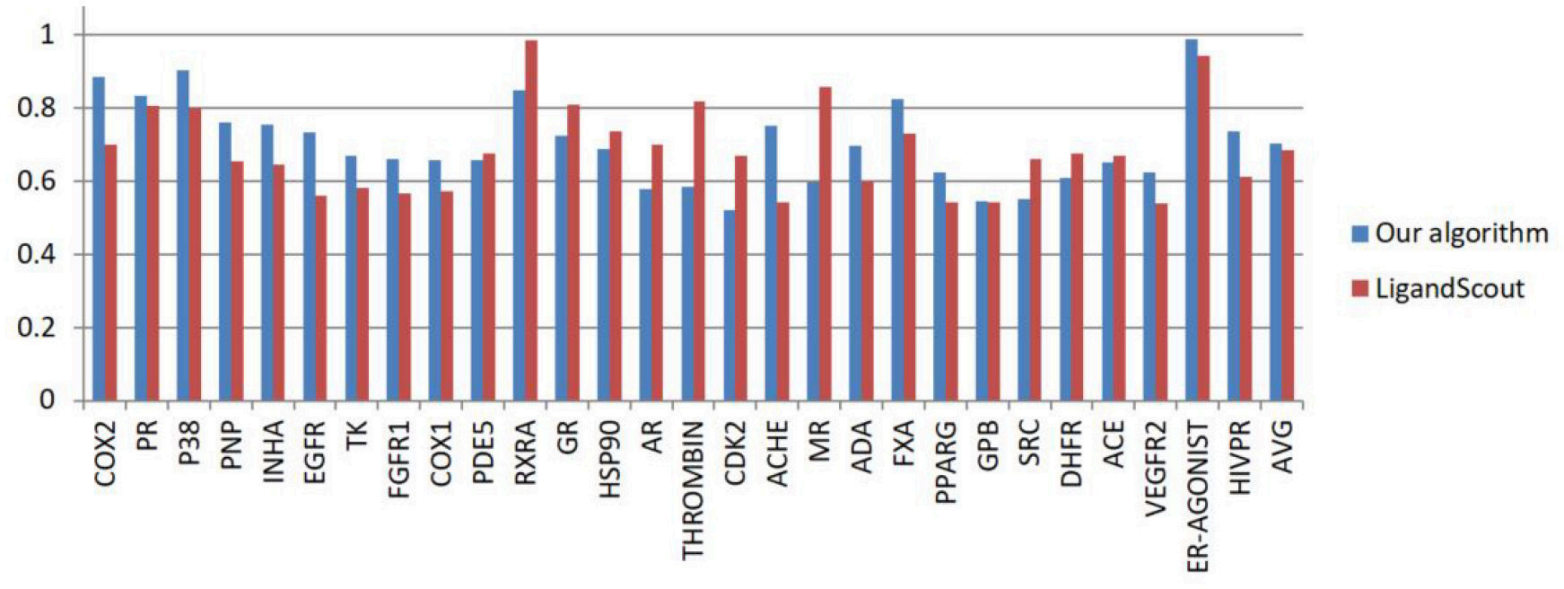
AUC values obtained by LigandScout and our algorithm from the 28 DUD datasets.

Using the 28 DUD datasets, pharmacophores were generated using our algorithm and LigandScout algorithm. Figure 7 shows the AUC scores calculated by the two algorithms. Detail information about DUD dataset and AUC computation for ER_AGONIST can be found from the supplementary materials.

As shown in Figure 7, the AUC value of our algorithm exceeds that of the LigandScout algorithm in 17 out of 28 test systems. The average AUC of our algorithm reaches 0.703, whereas that of LigandScout algorithm is 0.686, indicating a remarkable progress in pharmacophore generation.

**Figure 8:**
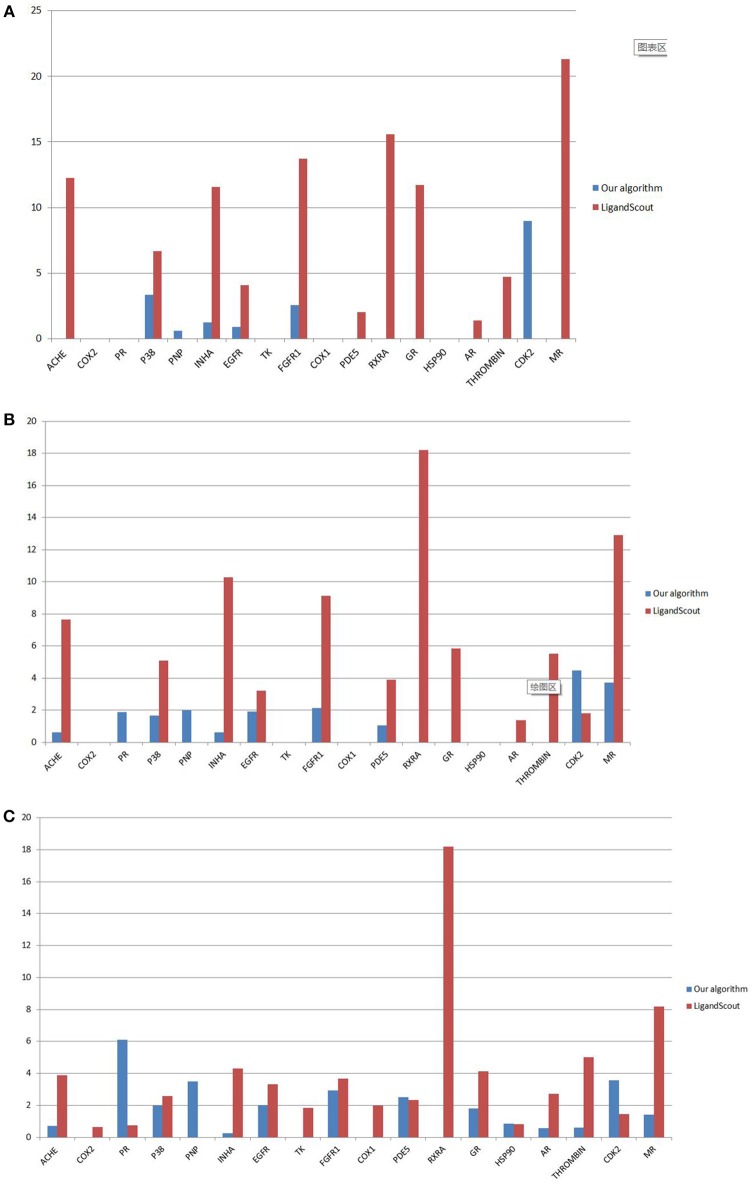
Comparison of EF values under 1% **(A)**, 2% **(B)**, and 5% **(C)** filter libraries.

Under 1, 2, and 5% filter libraries, the EF value of our algorithm is generally lower than that of LigandScout algorithm (Figure 8). This discovery illustrates the characteristics of our pharmacophore-extracting algorithm.

1. The pharmacophore model is not built on the ligand interaction but only from the receptor to deduce all the possible binding models. Hence, this design features universality.
2. The early enrichment effect is poor, but the whole active molecule can be effectively screened.

By analyzing and comparing the screening results through the AUC, we observed that the effectiveness of our pharmacophore model with apo protein structure surpasses that of related algorithms with holo protein structure on most datasets.

## Conclusion

This study designed and developed a new algorithm for pharmacophore extraction. This algorithm exploits the concept of SP hybridization and adds the aromatic probe and the 3 Å distance constraint to the original Pocket v.3 algorithm. The experimental results show that the pharmacophore extracted by the proposed algorithm features less distribution of pharmacophore feature points and high accuracy, thus enriching the current research on pharmacophore extraction without ligand information.

Additional future works are headed for this direction. Pocket size is an essential parameter in pharmacophore generation. When the pocket size measures more than three times of the average ligand volume, the number of feature points from the pharmacophore model increases significantly. It is necessary to further explore the reason why EF index is low and find a good solution. At the same time, the performance highly depends on the output quality of Cavity 1.0.

## Author Contributions

GH is the group leader and he is responsible for the project management and in charge of revising this manuscript. BG, JL, and YS are in charge of the data analysis and the planning and performing the experiments. SL designed the experiments and gave professional descriptions. XL provided valuable advice about this manuscript.

### Conflict of Interest Statement

The authors declare that the research was conducted in the absence of any commercial or financial relationships that could be construed as a potential conflict of interest.
