# Supplementary material for: An Improved Receptor-Based Pharmacophore Generation Algorithm Guided by Atomic Chemical Characteristics and Hybridization Types
Source: Front Pharmacol. 2018 Dec 17;9:1463. doi: 10.3389/fphar.2018.01463 (PMC6305075; doi:10.3389/fphar.2018.01463)
Supplement: Supplementary file 1 [file Table_1.DOCX]

| protein | PDB code | resolution (Å) | no. of ligands^b^ | no. of decoys | EFmax | EF1 | EF20 |
| --- | --- | --- | --- | --- | --- | --- | --- |
| Nuclear Hormone Receptors | | | | | | | |
| AR 1xq2 1.9 74 (a,b) 2630 60.2 33.5 3.8 ERagonist 1l2i 1.9 67 (a-c) 2361 29.6 19.2 4.5  GR 1m2z 2.5 78 (a) 2804 31.7 8.9 1.4 MR 2aa2 1.9 15 (a) 535 330.0 46.2 3.7 PPARg 1fm9 2.1 81 (a) 2910 1.0 0.0 0.0 PR 1sr7 1.9 27 (a) 967 2.9 0.0 2.0 RXRa 1mvc 1.9 20 (a) 708 148.5 24.8 2.2 Kinases CDK2 1ckp 2.1 50 (e,f) 1780 19.8 13.9 1.4 EGFr 1m17 2.6 416 (g) 14914 3.8 2.1 2.4 FGFr1 1agw 2.4 118 (g) 4216 1.0 0.0 0.2 HSP90 1uy6 1.9 24 (h) 861 10.8 8.6 2.0 P38MAP 1kv2 2.8 234 (g) 8399 4.1 2.1 2.4 SRC 2src 1.5 162 (g) 5801 3.1 1.2 1.5 TK 1kim 2.1 22 (a,d,i) 785 63.0 54.0 5.0 VEGFr2 1vr2 2.4 74 (j) 2647 2.2 1.3 1.4 Serine Proteases FXa 1f0r 2.7 142 (e,f,k) 5102 34.9 14.6 3.8 thrombin 1ba8 1.8 65 (e,l,m) 2294 18.3 13.7 2.9 Metalloenzymes ACE 1o86 2.0 49 (a,m) 1728 141.4 40.4 3.7 ADA 1stw 2.0 23 (a,e) 822 21.5 12.9 2.4 PDE5 1xp0 1.8 51 (f) 1810 29.1 11.8 2.3 Folate Enzymes DHFR 3dfr 1.7 201 (m) 7150 28.7 21.7 3.5 Other Enzymes AChE 1eve 2.5 105 (a,e,m) 3732 3.1 1.9 2.0 COX-1 1p4g 2.1 25 (i) 850 9.9 4.0 1.6 COX-2 1cx2 3.0 349 (c,f,m) 12491 29.1 20.1 3.3 GPB 1a8i 1.8 52 (e,m) 1851 28.6 22.8 4.1 HIVPR 1hpx 2.0 53 (a,e) 1888 9.3 3.7 2.2 InhA 1p44 2.7 85 (r) 3043 1.0 0.0 0.3 PNP 1b8o 1.5 25 (e,t) 884 158.4 31.7 4.4 | | | | | | | |
